# Intimate partner violence among women with mental health-related activity limitations: a Canadian population based study

**DOI:** 10.1186/1471-2458-14-51

**Published:** 2014-01-18

**Authors:** Janice Du Mont, Tonia Forte

**Affiliations:** 1 Women’s College Research Institute, Women's College Hospital, 7th Floor, 790 Bay St, Toronto ON M5G 1N8, Canada; 2 Dalla Lana School of Public Health, University of Toronto, 6th floor, 155 College St, Toronto ON M5T 3M7, Canada

**Keywords:** Intimate partner violence, Mental health, Activity limitations, Prevalence

## Abstract

**Background:**

There is strong evidence that women with serious or chronic mental illness experience higher rates of violence than women in the general population. Our objective was to examine the risk of intimate partner violence (IPV), a form of violence that is often recurrent and linked to negative physical and psychological consequences, among a representative sample of non-institutionalized women with activity limitations (ALs) due to a mental health condition.

**Methods:**

Data from the 2009 General Social Survey were used, a national, population-based, cross-sectional survey. The sample included 6851 women reporting contact with a current or former partner in the previous five years, of whom 322 (4.7%) reported a mental health-related AL always/often or sometimes.

**Results:**

The prevalence of any type of IPV was highest among women with mental health-related ALs always/often (54.4%), followed by women reporting ALs sometimes (49.9%), and those reporting no ALs (18.3%, p < 0.0001). The same pattern was observed for emotional (51.1%, 45.5%, 16.3%, p < 0.0001) and financial IPV (18.1%, 9.5%, 4.0%, p < 0.0001). For physical/sexual violence, rates were similar among women reporting mental health-related ALs always/often and sometimes, but were lower among those reporting no ALs (20.2%, 20.9%, 5.9%, p < 0.0001). In a logistic regression analysis the odds of having experienced any IPV remained greater for women reporting ALs always/often (OR = 3.65; 95% CI: 2.10, 6.32) and sometimes (OR = 3.20; 95% CI: 2.15, 4.75) than those reporting no ALs. Several social capital variables, including perceptions of having experienced discrimination, a weak sense of belonging in their local community, and low trust toward family members and strangers were also significantly associated with having experienced IPV.

**Conclusion:**

Findings suggest that women with mental health-related ALs may be at increased risk of IPV. Health and social service providers may need, therefore, to better target prevention and intervention initiatives to this population.

## Background

There is strong evidence that women with serious or chronic mental illness experience higher rates of violence than women in the general population [1,2]. A review of 11 studies that focused on serious mental illness and victimization found that women had a 13 to 19 fold increased risk of experiencing any violence compared to women in the general population [1]. Women with mental health problems may be at increased risk for violence for a number of reasons, including homelessness, histories of childhood abuse, drug and alcohol abuse [3,4], neurocognitive impairments that interfere with ability to evaluate danger, social skill and problem solving deficits, stigma, and social isolation [5].

Similar to their increased odds of experiencing other types of victimizations, women with mental health problems are at heightened risk for intimate partner violence (IPV) [6-8], a form of violence that is often recurrent and linked to substantive negative short- and long-term physical and psychological consequences [9-12]. Several review articles have summarized findings on the prevalence of IPV among women with mental illness, most of which have relied on clinic-based, nonrepresentative samples. In a review of 17 such studies published between 1966 and 2004 of women diagnosed with major depressive disorder, schizophrenia, schizoaffective disorder, or bipolar disorder, the prevalence of IPV ranged from 21% to 70% [13]. Another review of studies published up to May 2008 found rates of IPV ranged from 15% to 92% among psychiatric patients in hospital or out-patient care [6]. A more recent review and meta-analysis of 42 studies to March 2011 found that, compared to women without mental disorders, there is a higher risk of experiencing IPV among those with depressive disorders, anxiety disorders, and post-traumatic stress disorder [7].

Previous work has shown that those with mental illnesses have long experienced stigma and discrimination [14-16]. Stigma and discrimination can increase social distance and lead to social exclusion [14,17,18]. Studies have found that social exclusion can reduce the likelihood that persons with mental illness will become employed or access health care services for treatment of their disorder [19,20] and can lead to poorer health outcomes [21-23]. In the context of IPV, increased social isolation from family and friends and diminished social support may put women with mental health-related ALs at greater risk for harmful or abusive relationships [24]. Therefore, when examining the risk of IPV among women with mental illness, it is important to explore the role of such social capital factors in their experiences of abuse.

A number of Canadian population-based studies have examined the prevalence of IPV among women with ALs. However, it is important to note that these studies have defined ALs as those stemming from both physical and/or psychological health conditions [25-27]. This may be problematic given that women with ALs due to mental health conditions specifically may experience a range of other issues that are associated with increased risk of IPV such as substance abuse and homelessness [4,5,28]. As a consequence, studies that have examined ALs stemming from physical and/or mental health problems simultaneously may have masked the true rates of, and risk for, IPV among women with mental health-related ALs.

The purpose of our study was to provide estimates of the prevalence of different types of IPV among a representative sample of non-institutionalized women with ALs compared to those without ALs due to a mental health condition. Among women whose daily activities were limited by a mental health-related condition, we also examined the risk of having experienced IPV by the severity of the AL. Finally, we examined the relationship of mental health-related ALs and social capital factors to IPV. These latter factors may be useful in understanding and preventing IPV for women with mental health-related ALs.

## Methods

Ethics approval for this study was provided by the research ethics board at Women’s College Hospital. Data from Statistics Canada’s General Social Survey (GSS) were used, a cross-sectional, national, voluntary survey that collects information on Canadian’s experiences of victimization, as described previously [12]. It is the only survey of self-reported data on experiences of victimization Canada-wide. The population surveyed included those aged 15 years or older living in private households in the 10 provinces. Respondents were interviewed by telephone by trained interviewers and were selected using a process of Random Digit Dialing. Provinces were divided into geographic areas and all phone numbers within an area had the same probability of being selected. Once a household was successfully contacted, an individual aged 15 years or older was randomly selected to be interviewed. Interviews were conducted between February and November 2009 and were administered in English or French. Of the 31,510 households that were selected, 19,422 usable responses were obtained, representing a response rate of 61.6%. Various quality assurance measures were implemented by Statistic’s Canada at every step of the data collection and processing phase to ensure accuracy of responses [29].

### Mental health-related activity limitations

The GSS uses the World Health Organization’s framework for defining disability which includes difficulties in executing all types of activities [30,31]. For the present study, women with ALs due to a mental health condition were those who stated that a mental condition limited the type of activities they could engage in [30], assessed by the following question: “Are your daily activities at home, work, school or any other area limited by a psychological, emotional or mental health condition?” Response categories included always/often, sometimes, or no. The severity of mental health-related ALs was conceptualized as the frequency with which the respondent’s daily activities were limited with responses always/often indicating more severe ALs and sometimes indicating less severe ALs.

### Sociodemographic characteristics

Sociodemographic characteristics of the GSS respondents examined in the present study included age in years (15–34, 35–54, 55 and older), marital status (married/common-law, widowed/separated/divorced/single), immigration status (Canadian-born, foreign-born), highest level of education achieved (high school graduate or less, more than high school), annual household income in Canadian dollars (0-$19,999; $20,000-$49,999; $50,000 or more), presence of children younger than 15 years of age living in the home (yes, no), frequency of religious attendance (once per week, less than once per week, not at all) and region of residence (Eastern Canada [Quebec, Atlantic provinces], central Canada [Ontario], and Western Canada [British Columbia, the Prairies]).

### Social capital characteristics

Social capital was assessed using the following five indicators in the GSS: “Would you say that you live in a welcoming community”? (yes, no), “How would you describe your sense of belonging to your local community”? (very/somewhat strong, very/somewhat weak) and “Of those relatives and close friends you feel at ease with, how many live in the same city or local community as you”? (none, one or more). “Using a scale of 1 to 5 where 1 means ‘cannot be trusted at all’ and 5 means ‘can be trusted a lot’, how much do you trust each of the following groups of people: people in your family? people in your neighbourhood? people you work with or go to school with [asked among those who indicated they were employed or in school] and strangers”? Responses 1 to 3 were grouped as low trust and 4 and 5 as high trust. Finally, respondents were asked: “In the past five years, have you experienced discrimination or been treated unfairly by others in Canada because of…” ‘ethnicity or culture?’, ‘race or colour?’, ‘religion?’, ‘language?’, ‘sex?’, ‘physical appearance?’, ‘sexual orientation?’, ‘age?’, ‘disability?’, or ‘some other reason?’ (yes, no).

### Intimate partner violence

Survey respondents were asked about their experiences of IPV by a current or former partner with whom they had had contact with in the preceding 5 years from the date of the survey. All respondents who were legally married or living in a common-law relationship during the time of the survey, or had contact with their former partner in the previous five years were asked the spousal violence questions. The survey measured physical and sexual IPV using a modified version of the 10 item Conflict Tactics Scale (CTS) developed by Murray A. Straus [32]. Physical IPV in the past five years was assessed by asking respondents whether a current or former partner had threatened to hit them; threw something at them; pushed, grabbed, or shoved them; slapped them; kicked, bit or hit them with a fist; hit them with something that could hurt; beaten them; choked them; or used or threatened to use a knife or gun on them. Sexual IPV in the past five years was assessed by asking respondents, “Has your partner or former partner forced you into any unwanted sexual activity by threatening you, holding you down, or hurting you in some way”?

Respondents were also asked about their experiences of emotional and financial abuse from their current or former partner. The questions measuring emotional and financial abuse were originally created for use on Statistics Canada’s 1993 Violence Against Women Survey [33]. Emotional abuse was defined as having occurred if a respondent answered affirmatively to at least one of the following statements about her partner/former partner’s behaviour: “tried to limit your contact with family or friends, put you down or called you names to make you feel bad, was jealous and didn’t want you to talk to other men or women, harmed or threatened to harm someone close to you, demanded to know who you were with and where you were at all times, and damaged or destroyed your possessions or property”. Financial abuse was measured by the question, “Has your partner prevented you from knowing about or having access to the family income, even if you asked”?

In the present study, any IPV was defined as having experienced one or more types of physical, sexual, emotional, or financial abuse. The severity of IPV was measured in terms of the number of different types of abuse experienced (i.e., one type of physical, sexual, emotional, or financial abuse versus two or more types of violence) [12].

### Analyses

Analyses were weighted according to Statistics Canada’s guidelines to ensure that the findings were representative of the Canadian population [29]. Women who indicated that a mental health condition limited their activities of daily living always/often and sometimes were compared to women with no ALs due to a mental health condition across sociodemographic and social capital variables. Women with AL always/often and sometimes due to a mental health condition were also compared to women with no ALs due to a mental health condition on the prevalence of emotional, financial, physical/sexual, and any IPV and, among those reporting any IPV, compared on the severity of the violence experienced. All analyses were conducted with a *χ*^2^ test for categorical variables.

To determine whether the risk of experiencing any form of IPV was associated with the presence and severity of ALs due to a mental health condition, we conducted a weighted multivariate logistic regression analysis. Based on the variable selection criteria set out by Hosmer and Lemeshow [34], variables that were significantly associated with any IPV at the p < 0.15 level in a bivariate analysis were included in the multivariate logistic regression analysis. To maintain model parsimony, only significant variables were retained in the final model. The Hosmer–Lemeshow *χ*^2^ test on an unweighted model was used to assess the model’s fit. All variables in the model were evaluated for multicollinearity. We used the c-statistic—equivalent to the area under a receiver-operator curve—to examine the discrimination ability of the final logistic regression model.

For bivariate and multivariate analyses, a p value of 0.05 was considered statistically significant. For the logistic regression model, we report odds ratios and 95% confidence intervals. For household income, the proportion of missing data was 17%. Therefore, an unknown/not stated category was included in the analysis of this variable in order to retain the sample size.

## Results

Among the 19,422 respondents in the GSS, 10,694 were women. A total of 6900 women reported having had contact with a current or former partner within the previous five years from the date of the survey. Of these women, 110 (1.6%) reported a mental health-related AL always/often, 212 (3.1%) a mental health-related AL sometimes and 6529 (94.6%) no mental health-related ALs. Information on whether the respondent had an AL due to a mental health condition was missing for the remaining 49 (0.7%) women. Thus, the final sample consisted of 6851 women.

There were differences in the sociodemographic characteristics of women who reported a mental health-related AL always/often, women who reported a mental health-related AL sometimes and women who reported no mental-health-related ALs. Compared to women reporting no mental health-related ALs, women reporting ALs always/often or sometimes were less likely to be married (89.6% vs 70.1% vs 76.2%, respectively, p <0.0001) and more likely to report low annual household income of $0 to $19,999 (4.0% vs 17.7% vs 14.5%, respectively, p < 0.0001) (Table 1).

**Table 1:** Sociodemographic characteristics of women reporting contact with a current or former partner in the previous five years, by severity of mental health-related activity limitation (AL) (weighted number and %)

	** AL Always/often**		** AL Sometimes**		** No AL**		**p value**
	**N**	**%**	**N**	**%**	**N**	**%**	
**Age group (in years)**							
15-34	11	11.4	39	20.3	1438	21.9	.11
35-54	57	61.7	88	46.4	3048	46.4	
55 and older	25	26.9	63	33.3	2080	31.7	
**Marital status**							
Married/common-law	65	70.1	145	76.2	5880	89.6	<.0001
Widowed/separated/divorced/single	28	29.9	45	23.8	686	10.5	
**Education**							
High school or less	25	26.6	56	29.4	1778	27.2	.84
> high school	68	73.4	134	70.6	4758	72.8	
**Annual household income**							
0-$19,999	17	17.7	28	14.5	260	4.0	<.0001
$20,000-$49,999	23	24.6	46	24.4	1295	19.7	
$50,000 or more	39	41.5	85	44.9	3870	58.9	
Unknown/not stated	15	16.2	31	16.2	1141	17.4	
**Immigrant status**							
Canadian-born	74	79.4	160	84.0	5091	77.8	.26
Foreign-born	17	20.6	30	16.0	1451	22.2	
**Children <15 in living in the household**	29	31.0	60	31.8	2355	35.9	.42
**Religious attendance**							
Once per week	20	22.0	37	19.6	1349	20.7	.44
< once per week	40	43.1	65	34.4	2658	40.8	
Not at all	32	35.0	87	46.0	2507	38.5	
**Region of residence**							
Eastern Canada	28	29.8	56	29.5	2066	31.5	.62
Central Canada	30	32.5	69	36.6	2510	38.2	
Western Canada	35	37.8	64	33.9	1990	30.3	

There were differences between the three groups with regard to social capital characteristics. Compared to women reporting no mental health-related ALs, women reporting ALs always/often were more likely to report that they did not live in a welcoming community (2.6% vs 14.2%, p < 0.0001) and they felt a weak or somewhat weak sense of belonging to their community (21.0% vs 38.0%, p = 0.0001) (Table 2). Women reporting ALs always/often or sometimes reported lower levels of trust toward certain individuals than women reporting no ALs, including family members (7.4% vs 10.6% vs 2.3%, respectively, p < 0.0001), neighbours (46.3% vs 45.4% vs 31.6%, respectively, p = 0.0001), and people they work or go to school with (45.3% vs 40.2% vs 20.9%, p < 0.0001). Finally women reporting mental health-related ALs always/often were more likely to report discrimination in the previous 5 years compared to those reporting no such ALs (44.4% vs 14.7%, respectively, p < 0.0001).

**Table 2:** Social capital characteristics of women reporting contact with a current or former partner in the previous five years, by severity of mental health-related activity limitation (AL) (weighted number and %)

	** AL Always/often**		** AL Sometimes**		** No AL**		**p value**
	**N**	**%**	**N**	**%**	**N**	**%**	
**Live in welcoming community**							
Yes	79	85.8	176	94.7	6352	97.4	<.0001
No	13	14.2	10	5.2	173	2.6	
**Sense of belonging in local community**							
Very/somewhat weak	33	38.0	57	30.9	1360	21.0	.0001
Very/somewhat strong	54	62.0	128	69.1	5103	79.0	
**Relatives/close friends at ease with live in same city/local community**							
None	15	17.7	29	16.1	726	11.5	.10
One or more	73	82.3	151	83.9	5588	88.5	
**Trust in others**							
Family							
Low	7	7.4	20	10.6	151	2.3	<.0001
Hi	86	92.6	170	89.4	6381	97.7	
Neighbours							
Low	43	46.3	86	45.4	2043	31.6	.0001
Hi	50	53.7	104	54.6	4424	68.4	
People at work/school							
Low	25	45.3	51	40.2	1031	20.9	<.0001
Hi	30	54.8	77	59.8	3898	79.1	
Strangers							
Low	-	-	-	-	-	-	
Hi	-	-	-	-	-	-	
Any discrimination*, past 5 years							
Yes	41	44.4	73	39.0	959	14.7	<.0001
No	52	55.6	114	61.0	5564	85.3	

-Suppressed due to small numbers.

*Includes discrimination because of ethnicity or culture, race or colour, religion, language, sex, physical, appearance, sexual orientation, age, disability, or some other reason.

**Figure 1:**
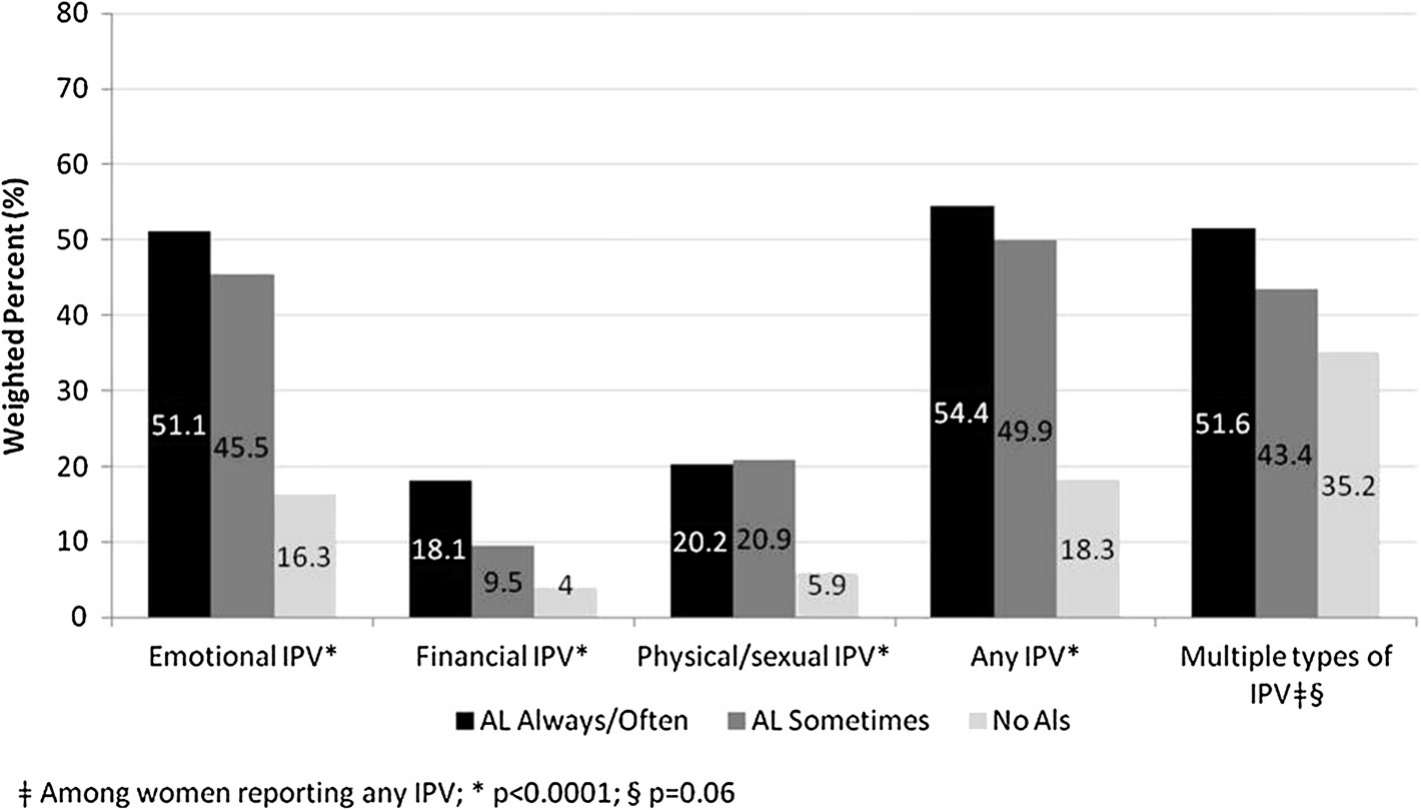
Prevalence and severity of intimate partner violence (IPV) among women reporting contact with a current or former partner in the previous five years, by severity of mental health-related activity limitation (AL) (weighted %).

The three groups also differed in the prevalence and severity of IPV experienced. Rates of any type of IPV, including emotional, financial, and physical and/or sexual, was highest among women with mental health-related ALs always/often (54.4%), followed by women reporting ALs sometimes (49.9%), and those reporting no ALs (18.3%, p < 0.0001) (Figure 1). The same pattern was observed for emotional abuse (51.1%, 45.5%, 16.3%, respectively, p < 0.0001) and financial abuse (18.1%, 9.5%, 4.0%, respectively, p < 0.0001). For physical/sexual violence, rates were similar among women reporting mental health-related ALs always/often and sometimes but were lower among those reporting no ALs (20.2%, 20.9%, 5.9%, respectively, p < 0.0001). Among women reporting any IPV, women reporting mental health-related ALs always/often (51.6%) and sometimes (43.4%) were more likely to report having experienced two or more types of violence compared to those with no ALs (35.2%), although this finding did not reach statistical significance (p = 0.06).

In the final logistic regression model (Table 3), compared to women with no mental health-related ALs, the odds of having experienced any IPV by a current or former partner remained greater for women reporting ALs always/often (OR = 3.65; 95% CI: 2.10, 6.32) and sometimes (OR = 3.20; 95% CI: 2.15, 4.75). Women reporting any discrimination (OR = 2.21; 95% CI: 1.81, 2.71), a weak sense of belonging in their local community (OR = 1.35; 95% CI: 1.11, 1.64), and low trust toward family members (OR = 2.79; 95% CI: 1.80, 4.31) and strangers (OR = 1.44; 95% CI: 1.05, 1.99) also had a higher risk of having experienced any IPV compared to women who did not. The odds of having experienced IPV was also higher among younger women (OR = 1.44; 95% CI: 1.13, 1.83, women aged 15–34 years), women who were widowed, separated, divorced, or single (OR = 8.76; 95% CI: 7.22, 10.62), and women reporting a lower annual household income (OR = 1.67; 95% CI: 1.17, 2.40, annual household income 0-$19,999).

**Table 3:** Weighted logistic regression analysis of factors associated with any intimate partner violence among women reporting contact with a current or former partner in the previous five years

**Factor**	**Odds ratio**	**Lower 95% CI**	**Upper 95% CI**
**Mental health-related activity limitation**			
Always/often	3.65	(2.10)	(6.32)
Sometimes	3.20	(2.15)	(4.75)
Never	1.00		
**Age group (in years)**			
15-34	1.44	(1.13)	(1.83)
35-54	1.47	(1.20)	(1.79)
55 and older	1.00		
**Marital status**			
Married/common-law	1.00		
Widowed/separated/divorced/single	8.76	(7.22)	(10.62)
**Annual household income**			
0-$19,999	1.67	(1.17)	(2.40)
$20,000-$49,999	1.38	(1.12)	(1.71)
$50,000 or more	1.00		
Unknown/not stated	1.03	(0.81)	(1.33)
**Any discrimination, past 5 years**			
Yes	2.21	(1.81)	(2.71)
No	1.00		
**Sense of belonging in local community**			
Very/somewhat weak	1.35	(1.11)	(1.64)
Very/somewhat strong	1.00		
**Trust toward family**			
Low	2.79	(1.80)	(4.31)
Hi	1.00		
**Trust toward strangers**			
Low	1.44	(1.05)	(1.99)
Hi	1.00		

Hosmer-Lemeshow *Chi-squared* = 8.97 (p = 0.25); c-statistic = 0.77.

## Discussion

Despite the growing body of research on IPV in the general population, there is little high quality evidence on the risk of IPV among women with ALs due to a psychological, emotional or mental health condition. This study showed that the prevalence of IPV is higher among women with mental health-related ALs compared to women reporting no mental health-related ALs. While these findings are among the first reported in Canada using a population-based sample, our findings are consistent with previous studies using clinic-based samples [6,7].

It has been suggested that the relationship between IPV and mental health-related ALs is likely bi-directional [7]. That is, mental health-related ALs may both precede or be a consequence of IPV. The impact of IPV on mental health outcomes has been well documented [35]. Research also shows that having a mental health-related AL is associated with experiencing subsequent abuse, [36] likely the result of the functional impairments that may accompany such ALs and that place these women at increased risk for homelessness, drug and alcohol abuse [3,4] and social isolation [5].

In both bivariate and multivariate analyses, our study showed a dose response relationship between the severity of mental health-related ALs, conceptualized as the frequency with which the ALs limited women’s daily activities, and the risk of IPV. The prevalence of all types of violence, emotional, financial, physical and/or sexual, was higher among women reporting mental health-related ALs always or often compared to those reporting ALs sometimes. These findings suggest that women with more severe ALs may be especially vulnerable to being victimized. Previous research shows that exposure to trauma among women already suffering from mental illness can lead to physical health problems, a worsening of the underlying psychological problems, psychiatric comorbidity, and poorer functioning and quality of life [4,37].

Even after adjusting for the presence of mental health-related ALs, this study identified a number of social capital factors to be associated with women’s experiences of IPV. Women reporting lower levels of trust for family were more likely to have experienced IPV. In addition, women with lower levels of trust for strangers and a poorer sense of belonging to their community were more likely to report having experienced IPV, although these associations were less strong. It is possible that lack of trust and feeling isolated from one’s community were the result of having experienced IPV. These results are consistent with previous work in IPV showing that increased social isolation from family and friends and diminished social support is a common feature of abusive relationships [24]. In our study, women with mental health-related ALs were more likely than those with no mental health-related ALs to report a weaker sense of belonging to their community, lower levels of trust toward family members, neighbours, and people they go to school/work with, and were less likely to report that they lived in a welcoming community. Therefore, given these circumstances, women with ALs may be at a particularly heightened risk for abuse. Together, these findings may have important implications for women’s help-seeking for IPV as it may prevent them from disclosing the violence to family members and others and may inhibit them from leaving a violent relationship [24].

Consistent with previous work linking perceptions of discrimination with IPV victimization and perpetration [38], we found that women reporting having experienced any discrimination were more likely to have experienced abuse. This finding linking discrimination to IPV may be of particular concern for women with mental health-related ALs. Our bivariate results showed that considerably more women with ALs reported discrimination than women with no ALs, with the percentage highest among women reporting more frequent ALs. Previous Canadian research has revealed that that over half (53%) of women who met the criteria for a mood or anxiety disorder or substance dependence reported having faced discrimination as a result of their mental health problems [39]. Perceived discrimination has been shown to be associated with depressive symptoms, generalized anxiety, psychiatric distress and general well-being among those in the general population [40,41]. For women already suffering from mental health-related ALs, the consequences of having experienced discrimination may be greater and may discourage women from seeking help for both the IPV and their mental health problems from social, health, and criminal justice services [42,43].

Also similar to findings of past studies, several sociodemographic characteristics of women were found to be associated with having experienced IPV. In particular, young women were more likely than older women to have experienced IPV [26,44] which may, in part, reflect that young women tend to form relationships with younger partners who tend to be more violent [45]. Women who were divorced, separated, widowed or single were more likely to have experienced IPV as were those who reported lower annual household income [26,46]. Financial strain and associated stress, which can accompany low socioeconomic status, can increase the risk of IPV and economic dependency on the abuser can inhibit women’s ability to terminate an abusive relationship [47,48].

A number of limitations to the current analyses must be noted. First, because this survey based study assessed mental health-related ALs concurrently with IPV, we were not able to assess whether IPV preceded or followed the development of ALs. Second, our study is based on self-reported experiences of IPV and AL. Given the sensitive nature of IPV and the stigma that may be attached to mental illness, respondents may have been reluctant to report both abuse and mental health-related ALs, resulting in an underestimation of the true extent of these issues. Finally, our study does not differentiate between the different causes of women’s mental health-related ALs and their relationship to having experienced IPV. A recent meta-analysis, however, showed a higher prevalence and an increased likelihood of being a victim of IPV among women across all diagnostic mental health categories, including depressive disorders, anxiety disorders and Post-Traumatic Stress Disorder [7].

## Conclusion

Our findings have several important implications for both practice and research. Although our study could not ascertain the directionality of the relationship between IPV and mental health-related ALs, our results show that compared to women with no such ALs, the prevalence of IPV is higher among women with mental health-related ALs. These findings suggest that prevention and intervention activities may need to better target these women so that the negative impacts of abuse for them can be ameliorated. Service provision and program planning require an understanding of the interconnection between violence and mental health. Increased collaboration between mental health services and women’s IPV services along the course of treatment could help address these concerns. Our findings support further investigation into the role of social capital factors in understanding the experiences of IPV among women with and without mental health-related ALs. Future research should also investigate whether IPV has a greater negative impact on the physical and psychological well-being of women with mental health-related ALs whose health is already compromised.

## Abbreviations

IPV: Intimate partner violence; ALs: Activity limitations.

## Competing interests

The authors declare that they have no competing interests.

## Authors’ contributions

JDM conceptualized the study and designed and oversaw its implementation. JDM and TF developed the study’s analytic strategy. TF helped conduct the literature review and both authors contributed to drafts of the manuscript. Both authors read and approved the final manuscript.

## Pre-publication history

The pre-publication history for this paper can be accessed here:

http://www.biomedcentral.com/1471-2458/14/51/prepub
